# “I am getting something out of this, so I am going to stick with it”: supporting participants’ home practice in Mindfulness-Based Programmes

**DOI:** 10.1186/s40359-020-00453-x

**Published:** 2020-08-31

**Authors:** Jiva Masheder, Lone Fjorback, Christine E. Parsons

**Affiliations:** 1 Mindfulness Brighton, Brighton, UK; 2 Danish Center for Mindfulness, Aarhus, Denmark; 3 Danish Center for Mindfulness, Department of Clinical Medicine, Aarhus University, Aarhus, Denmark; 4 Interacting Minds Center, Department of Clinical Medicine, Aarhus University, Aarhus, Denmark

**Keywords:** Home practice, Treatment engagement, Mindfulness, Homework, Meditation practice

## Abstract

**Background:**

The practice of mindfulness at home is a core component of standard eight-week mindfulness-based programmes (MBP). Teachers of mindfulness courses require an understanding of the factors that need to be addressed to support participants in establishing and maintaining a mindfulness practice.

**Method:**

Here, we present a review of seven factors that we argue are important for participants’ practice of mindfulness. We use the well-established model of Behaviour Change, the COM-B model (Capability, Opportunity, Motivation and Behaviour) to organise and consider these factors. For each factor, we first present a definition and then a discussion in relation to psychological, health and Buddhist literature. We illustrate the importance of each factor with quotes from MBP participant interviews.

**Results:**

We discuss participants’ Capability (planning/commitment, physical space), Opportunity (social support, the relationship with the teacher) and Motivation (readiness for self-care, beliefs about practice, self-efficacy, experiencing the rewards of practice), and how these lead to the target Behaviour (mindfulness practice).

**Conclusions:**

Our understanding, as teachers and researchers, of how best to support and guide participants during MBPs is at an early stage. We draw out practical lessons around each of the seven factors for mindfulness teachers in supporting participants’ home practice.

## Background

How can mindfulness teachers best support their class participants in establishing and maintaining a mindfulness practice? Mindfulness practice, like other new behaviours (e.g., engaging in physical activity, or sleeping sufficiently) can be difficult to start. Even if someone starts a regular practice, maintaining this new behaviour can be challenging. Understanding how to help participants establish a regular mindfulness practice, and integrating this into a sustainable daily routine, is therefore an important question for many mindfulness teachers and practitioners.

There is now a robust body of evidence indicating that eight-week Mindfulness-Based Programmes (MBPs) such as Mindfulness-Based Cognitive Therapy (MBCT) and Mindfulness-Based Stress Reduction (MBSR) courses can lead to improvements in mental and physical health (meta-analysis, see [26]). Meta-analytic evidence indicates that MBPs are more effective than non-active or active control conditions, and generally equivalent to other evidence-based treatments that require similar behaviour change. Within MBPs, regular practice of mindfulness at home is argued to be fundamental to becoming aware of, and relating differently to, mental habits and behaviour patterns and ultimately to behaviour change [17, 41, 63]. Home practice consists of both informal and formal mindfulness exercises that train attention and develop the ability to respond to difficult mental and physical experiences [41]. Both formal and informal practice require effort and sustained commitment from participants.

While research examining the relationship between mindfulness practice and a range of outcomes is still in its infancy, a recent meta-analysis reported that the overall extent to which participants report engaging in formal home practice correlates with overall treatment changes [55]. Interestingly, this review indicated that the amount of practice that participants report completing is variable, with participants on average completing around 64% of assigned amounts (around 30mins per day, 6 days per week, 95% CI 60–69%). There was a significant, albeit small, relationship between self-reported home practice completion and treatment benefit (see also systematic review by [47]). Given the importance of mindfulness home practice, we explore the factors that affect whether and how practice becomes an established, sustainable part of participants’ daily routines during MBPs. This is intended both to inform mindfulness teachers and to provide a guiding framework for future research.

## Methods

We chose a theory-building approach to the question “What supports establishing and sustaining a mindfulness practice?” We drew on a broad and diverse set of sources. Our sources were: (1) the psychological literature on health behaviour change, focusing on factors relevant to mindfulness practice; (2) the mainstream secular mindfulness literature providing guidance on mindfulness practice; (3) ancient and more contemporary Buddhist texts from teachers and practitioners in the field, which discuss issues around mindfulness practice and; (4) interviews with MBP participants. Our approach was informed and shaped by our experiences as mindfulness practitioners, teachers and researchers. In reviewing the literature, we took a narrative approach, selectively considering highly-cited articles from the fields of behaviour change, mindfulness and Buddhist literature. We used the key terms ‘home practice’, ‘practice’, ‘mindfulness’ ‘Mindfulness-Based Stress Reduction, ‘Mindfulness-Based Cognitive Therapy’, ‘engagement’, ‘compliance’ and ‘adherence’.

Interviews were conducted by JM and participants were drawn from general public groups. The participant age range age was from 28 to 60 years and 6 were female and 3 were male. The interviews with course participants were at three points, halfway through (4 weeks), at the end of the programme (8 weeks), and two to 3 months after the programme finished. One person dropped out after 3 weeks, so only one interview was conducted with this individual. The interviews were recorded and permission was obtained to use quotes for this article, which is based on a Master’s thesis conducted by JM, as part of the MSc in Mindfulness-Based Cognitive Therapies at the University of Exeter, UK (overlapping material is included from this thesis). Quotes were chosen to illustrate points derived from the literature and are not considered to be a data primary source. The 25 interviews with the MBPs participants started with open-ended questions (What helped/hindered you in developing and sustaining a mindfulness practice?) and then developed into a more in-depth dialogue to understand the factors that were key for those individuals.

The iterative process of a focused literature review, an examination of participant interviews and dialogue among the authors took place over 2 years. We note that the present review is not intended as a systematic literature review, nor as primary qualitative research, rather a discussion of factors around mindfulness practice that we consider to be key. Finally, we use one widely-established model, the COM-B model (Capability, Opportunity, Motivation and Behaviour; [52]) as an organising framework to consider the factors identified through the literature review. These factors can be examined further in qualitative research, with a larger-scale approach to participant interviews, and with a systematic literature search.

### Practice in mindfulness-based Programmes (MBPs)

We begin with a definition of mindfulness practice, which takes a number of forms in MBPs. Mindfulness practice includes ‘formal’ exercises, such as the body scan, mindful movement and sitting mindfulness, lasting typically around 45 min (some studies have reported using reduced practice times, see [55]). Practices can also be shorter, such as the three-minute breathing space. MBPs also encourage informal practices, bringing mindfulness into everyday life with activities that the participant is doing already. Examples include mindful eating, mindful routine activities, and mindful walking. Both formal and informal practice are argued to be fundamental to learning within MBPs. We refer to practice, including both formal and informal, but note that the majority of research to date has centred on longer, formal practice. In this article, we focus on participants’ practice during the 8 weeks of an MBP because this is when teachers are in face-to-face contact with participants, and also there is the largest evidence -base from MBP studies.

### What supports and sustains mindfulness practice?

We propose seven factors that can be organised into an explanatory framework using one of the well-established models of behaviour change, the COM-B model (‘Capability’ – psychological or physical skills or knowledge, ‘Opportunity’, ‘Motivation’ and ‘Behaviour’). Within the COM-B model, Capability is defined as the individual’s psychological and physical capacity to engage in a specific behaviour. Motivation is defined as all of the brain processes that energize and direct behaviour, and includes habitual processes, emotional responding, as well as analytical decision-making. Opportunity is defined as all of the factors external to the individual that make the behaviour possible or prompt it. The model suggests that the dynamic and synergistic interaction between Capability, Motivation and Opportunity brings about both the learning of a new Behaviour, and also sustains the Behaviour over time. We have selected this model because it is designed to provide a comprehensive conceptual framework of behaviour [52], and has been applied to numerous health-related behaviours such as physical activity [36]) and dietary changes [6].

The seven proposed factors that support and maintain mindfulness practice are: (1) self-efficacy; (2) self-care; (3) beliefs about practice; (4) planning/commitment; (5) social support; (6) the relationship with the teacher and; (7) experiencing the rewards of practice. We organise these factors in terms of the COM-B model, grouping the factors into the three overarching domains of Capability (planning/commitment, physical space), Opportunity (social support, the relationship with the teacher) and Motivation (self-care, beliefs about practice, self-efficacy, experiencing the rewards of practice) leading to the target Behaviour (mindfulness practice). Within this model, each domain influences whether mindfulness practice is learned and sustained. Furthermore, Opportunity-related factors might influence Motivational factors, and so impact the Behaviour, formal mindfulness practice. We begin by defining each factor, before describing how it is represented in the secular and Buddhist mindfulness literature. Each factor is considered with reference to relevant literature and illustrated with quotes from MBP participant interviews. Finally, we discuss practical strategies that mindfulness teachers may adopt in their classes to target each factor (e.g., how might we increase participants’ self-efficacy?).

### Capability: participants’ planning and commitment to practice

Setting goals and making plans are common ways of helping us to modify a health behaviour. In the context of MBPs, this requires participants to plan and commit to both formal practice time and informal practices. The language around planning has to be carefully constructed in a mindfulness training context. Participants are invited to let go of striving and instead connect with a meaningful commitment to mindfulness practice. This practice is present-moment oriented and includes an element of acceptance of what arises. It is also helpful to differentiate between behavioural goals and outcome goals. Setting the goal of completing a formal practice is a behavioural goal, whereas setting a goal such as ‘I will feel relaxed at the end of this practice’ is an outcome goal, which we avoid in MBPs.

Building on behavioural goals, participants can use implementation strategies to move from general plan of ‘I will do regular mindfulness practice’, to specifying times and places. Considering the COM-B model, the capacity to plan is positioned as a psychological ability required to engage in the behaviour of practice. More generally, participants’ capability is discussed in the initial orientation session of an MBP, where there is a consideration of any relevant current conditions (e.g., a major depressive episode) or life events (e.g., recent bereavement), as well as how realistic the time commitment is for participants during the course period.

Action plans, specifying where and when to practice, have shown promise in increasing the frequency of participants’ at-home formal practice [24]. This might also be applied to informal practices (e.g., ‘I will prepare my vegetables mindfully and with awareness’). An ‘if-then plan’ is an evidence-based strategy to help people get started and continue with a health goal [27, 28, 30]. Such plans set structure in which people (i) note opportunities for, or barriers to, taking action, (ii) identify a response to each opportunity and barrier and then (iii) establish a connection between the opportunity or barrier and the response [60]. Meta-analyses have found medium to large effects of ‘if-then plans’ on goal achievement across a wide area of behaviours [13, 30].

Implementation plans may only be necessary when the target task is challenging [65]. Some participants find practice more challenging than others; these may be the participants who need to give themselves this extra support and structure. Most participants have phases when practice is easier and phases when it is harder, and during those times when it is more difficult, a plan could be a helpful support. Bartley [11] describes making action plans for difficult times which include doing the course again alone, phoning a friend for support, and keeping up a daily practice. Similarly, Burch [16] suggests that participants prepare a personal ‘first-aid kit’ of the practices they find most useful for when motivation is low.

By week 4, Suzanne had established a plan to support herself: ‘having that regular time and I’ve stuck with it, putting the body scans in place…generally when I came home after work, around 6, in my bedroom, with a mat that I lay down with, …having a good regular practice.’ ‘having a structured approach is better for me than being left to my own devices although that’s not what I grew up with’. When she tried to practice without that supporting structure, it was more difficult. Conversely, without a plan or routine, Lucy found it hard to re-establish a practice after her initial success with it, and then found competing priorities took over (post-course): ‘I found I was thinking about it too late in the evening – it’s so hard to break habits…During the course I used to practice straight after putting my daughter to bed – that worked for a while but then the routine was broken by builder chaos and I didn’t manage to get into the routine after that…it’s easier to put a DVD on.’

### How to support planning and commitment to practice

We suggest that helping participants to form a definite plan of when they will carry out their practices may be useful, although we need to be careful that it does not become yet another task-oriented ‘chore’. Georgie was aware of this possibility at week 4 - ‘I don’t want to set up too many things that I have to do’. Practice may instead be presented as an act of self-care, rather than a demand on participants’ time. Sumedho [70], a senior Buddhist monk and former Abbot (1984–2010) at the Amaravati Buddhist Monastery, also discusses this, encouraging us to see practice as important as sleeping and eating, rather than another duty or burden, and letting us know that eventually, it becomes something to look forward to, as an opportunity to get out of mental ruts.

Having a clear commitment can also reduce time spent wondering about whether to do the practice or not [29]. As Georgie said at week 4 ‘It’s useful to pre-emptively make decisions, it saves time.’ Similarly, and at the same stage, Zoe said ‘If there’s a moment’s hesitation then I remember my commitment.’ Conversely, Liam had not made that clear commitment and struggled to establish a practice. Among the interviewees, it was clear that those who had established a routine or clear ‘if-then plans’ found them highly supportive. Those who felt they needed to, but had not managed to, then struggled to practice regularly despite their best intentions. For almost everyone, making a plan is likely to be helpful as it means the decision is ‘already made’, a phrase that came up in several interviews.

Another factor in Capability is that of physical capability. While people with any level of physical capacity can practice mindfulness – indeed Kabat-Zinn’s first participants were the chronically and terminally ill, as described in the foundation text for all MBPs [41] - having a space in which to practice is important for formal, guided meditation practices. Two interviewees reported a lack of their own space as an obstacle to doing the longer practices. As teachers, we can encourage participants to think about how they can create an uninterrupted space. This might include asking partners or friends for support with children and pets, and thinking about where they can do the practice without external disturbance. This may be worth discussing in Week 1: where, when and how to do the home practices, and how to work with potential obstacles. It is also helpful for participants to explain what is involved in doing the course to their family so they can get the necessary space and support.

### Opportunity: social support

Social support can be defined as an individual’s perception or experience of affection, care, value, belonging, or assistance in connection with others or networks of others [71]. The stress-buffering effect of social support is arguably amongst the most tested impacts of social relationships on health [34]. Perceived levels of social support have been associated with numerous physical health markers, especially in mid-adulthood, in large-scale life span analyses [77]. Social support falls within “Opportunity” in the COM-B model, whereby the individual’s physical (see above) and social environment can facilitate or prompt practice behaviour.

Social support has been found to be correlated with people’s engagement in a range of health behaviours such as physical exercise [23] and healthy eating habits [23]. A recent meta-analysis provided evidence for a robust association between social support and positive sleep outcomes [43]. Furthermore, improving social support may also improve health outcomes: a 2019 meta-analysis reported that interventions directly targeting social support networks had significant, positive effects on a range of health behaviours and health-related outcomes [37]. Social support, in various forms such as family and the group setting, is likely to be important in MBPs as well. Most people learn mindfulness practices in a peer group, and that group can provide significant support.

Contemporary Buddhist teachers also emphasise social support. For example, Thich Nhat Hanh [33], global spiritual leader and Zen teacher who in 1966 founded the Buddhist Order of Interbeing at Plum Village in France, says ‘The presence of those who practice mindful living is a great support and encouragement to us…getting in touch with an existing sangha [community] or setting up a small sangha amount to a very important step’ (p.146 [33];). Similarly, Ajahn Sucitto, former Abbot of the first monastery of its lineage in the UK (1992–2014) the Cittaviveka Buddhist Monastery says: ‘Meditating with a few friends at regular times can be a great support towards constancy of practice’ (p. 22 [67];).

Indeed, the importance of social support in establishing and maintaining a meditation practice was recognised as far back as the original Connected Discourses (Samyutta Nikaya), where the Buddha stresses the importance of companions and friends on the path of practice [14]. Central to Buddhist practices are the ‘Three Refuges’; the word ‘refuge’ is used to denote a safe metaphorical ‘place’ to practice and come to understand the causes of our stress. The first refuge is in the Buddha – either in the teacher or in the qualities of wisdom and clear-sightedness within the teacher. The second refuge is in the Dharma – the teachings and practices that lead to wisdom, which can also be understood as ‘the truth of the way things are’. By observing phenomena in our mindfulness practice, we come to understand features like impermanence and the futility of clinging to what will inevitably change, and the painful pointlessness of resisting our experience. The third refuge is in the Sangha – the spiritual community as a necessary support for the practice [4].

In secular mindfulness literature, Santorelli [62] also highlights the power and emotional support offered by the group: ‘Thirty strangers. Thirty different reasons for being here. Yet in our differences, we are drawn together around a common intention: to learn to care for ourselves and be alive to our living; to look deeply into our own lives and to do so collectively.’ He then goes on to say, ‘My mother died during the fourth week of class. Often, I felt deeply nourished by the caring offered, mostly in silence, by the patients, as if I were their patient’ (p. 47).

Several interviewees remarked at weeks 4 and 8 how supportive they found the group, sharing different aspects of their group experience. This is consistent with other qualitative analyses (e.g., [49]). Noeleen articulated this in the first interview at week 4: ‘It’s good to have the group participation and to hear their experiences, though I’m not getting it yet.’ Lucy found one aspect of the group particularly helpful, as she remarked at week 8: ‘There is something about reporting it back, if we weren’t doing that it would be easy to slip’.

However, a group is not always experienced as supportive; social norms are only helpful if people identify with the others [72]. Georgie found this when she looked for a group after the course had finished: ‘In the [original] group, it was nice to feel you’ve got company in your endeavour to do something good for yourself – I didn’t feel that connection with any of the people there [at the other group]– they seemed a little stand-off-ish, I didn’t feel we had much in common’. Similarly, other qualitative studies have reported that some individuals have difficulties with the group setting (e.g., [35, 49]) and it can be a reason for drop out from courses [61]. Eleanor found at week 4 that ‘the group isn’t always supportive – people saying ‘it’s not working for me’.

Of course, the original MBSR or MBCT group lasts only for 8 weeks and is a small time period within people’s lives. Participants are therefore likely to benefit from other forms of social support. Friends and partners can be helpful. Meichenbaum & Turk [51] describe involving family, but needing to be sensitive to the dynamics within the family; if the practice takes time away from other activities the participant usually does, s/he may meet with resistance from partner or family which will need to be resolved. A buddy system may also be useful, as Kathryn said at week 4: ‘I have a friend who had done the course so we became practice buddies, some kind of joint responsibility…I can convince myself I don’t need to actually do it but I wouldn’t lie to her. In a group, it’s easier to stay quiet’.

Seeking social support can also help maintain practice when it becomes difficult, as Bartley [11] suggests, as can seeking out talks, classes and group sittings [41]. Several interviewees expressed in the first two interviews that they found the group supportive. For most, the end of the 8-week course was a major transition, with several participants saying things like ‘I was worried when the course finished it would all fall apart’ (Suzanne, post-course). Several interviewees expressed an intention to get back in touch with the rest of the group after the course had finished, and several had investigated mindfulness groups and yoga classes near them. Forms of social support are likely to change over time as people’s needs and circumstances change. The increasing availability of online virtual meditation classes and meetings offers an opportunity for people who may be physically isolated, for reasons of health or geography, to practice and discuss their experiences with others.

What characteristics are necessary for the group to be supportive? As teachers, part of our role is to create that safe space for participants. Factors that contribute to this can include group boundaries, both physical (space, timekeeping, absences) and internal (acceptable behaviour and speech, [11]). We can also provide a safe space by modelling the attitudes of non-judgement and acceptance [50]. Pair and small group work can also help the group to bond, as participants are likely to feel less exposed talking to one or two others than in front of the whole group, and may share more personally. By sharing their experiences, participants can create a social norm of support, as Zoe found (week 8): ‘I found the group supportive and [the teacher] supportive and it was good to hear other people’s experiences – the problems are shared and more or less the same for everyone.’

A supportive environment within the 8-week course group, at home and post-course, are all helpful, though insufficient for mindfulness practice. As teachers, we can foster a supportive group by setting group boundaries and norms to make it a safe space, and also make sure participants know in advance the level of home practice that will be assigned so they can try to get support from their partners and families. Teachers may also offer follow-up sessions and can signpost participants to local groups, classes and relevant reading as they finish the course.

### Opportunity: participants’ relationship with their teacher

All psychological therapies place an emphasis on the importance of the therapeutic relationship, and this is also the case for MBPs. For individual psychotherapy, meta-analytic evidence indicates a robust, predictive association between the therapeutic alliance and therapy outcomes [22]. However, establishing a causal role for the therapeutic alliance in outcomes is methodologically challenging and studies to date have been observational, and have not attempted to directly manipulate the therapeutic alliance [19].

Nevertheless, the therapeutic alliance is championed by many as the most important ‘common factor’ across psychotherapies. In MBPs, teachers’ modelling of the seven attitudinal foundations of non-judging, patience, beginner’s mind, trust, non-striving, acceptance and letting go is crucial for participants [41]. Salmon et al. [61] discusses how teachers should call or email any participant who misses a class to see how they are and keep them up to date with the course. This is to show the participant that the teacher cares about them and to help them to continue with their practice. The relationship with the teacher again falls into the category of social Opportunity within the COM-B model. According to McCown et al. [50], ‘the teacher’s authentic presence reciprocally supports participants’ capacities to not know…and to stand with/in their own experiences, which may lead to insights’.

The Thai Buddhist teacher, Buddhadasa Bhikkhu, who was highly influential in the development of Western mindfulness practice, used this approach too. One of his pioneering students who brought Buddhism to the West, Kornfield [45] says, ‘he has [students] come and sit next to him and treats them as a spiritual friend, engaging in warm-hearted conversation and enquiry, encouraging students to respect themselves and their own vision.’ (p.233).

Georgie certainly found this important at week 8, saying how she appreciated the MBP teacher’s gentle forgiving approach; his attitude was very congruent with the approach of gentleness and forgivingness – he modelled the attitudes well. ‘A nice naivety and openness – there was never a sense that you asked a stupid question’.

The teacher’s own engagement with mindfulness practice is also crucial. The teacher needs to model the attitudes that are cultivated in the practice, and to have sufficient commitment to the home practice to inspire the participants [15]. It is also important that the teacher has experience of working through the difficulties involved in practice; how else can s/he guide others? McCown et al. [50] describe it thus ‘the teacher has been there, known it…she has sat with her own loss, her own pain, her own anger, and can speak from those truths.’ (p.116).

Meichenbaum and Turk [51] also discuss how the teacher can support participants. The teacher needs to help the participants consider barriers to practicing and note any specific problems, and be realistic about what is possible in their lives at a given time. They also need to monitor whether the practice is being carried out (as recommended by [63]). and how participants feel about it. This is one of the purposes of the Enquiry phase of each class session: it is important to ensure that each participant has the opportunity to speak about their home practice each week, whether in the full group or in pairs.

As teachers, our relationship with the course participants can influence the likelihood of them practising or not. We need to be receptive, present, aware and listen to each participant with interest and care to help them feel that their learning is a valuable and collaborative process. We may also find it helpful to reveal aspects of our own practice, being careful that it is relevant for participants [15, 50]. Letting participants know that we too engage in these practices and sometimes find them difficult may well be supportive. Some form of informal contracting may help as well, in discussing and formulating practice plans.

### Motivation: experiencing the benefits of mindfulness practice

Internal (intrinsic) rewards play a role in establishing a practice or habit of any kind: we are more likely to maintain a health behaviour if we are satisfied with the outcome or results. For physical activity, experimental evidence suggests that specifically targeting feelings of enjoyment during exercise can increase activity adherence [39]. Across psychological therapies, early symptom improvement has been identified as a strong indicator of overall treatment outcome across a range of disorders (e.g., [48, 73]). Such findings suggest, albeit indirectly, that experiencing the benefits of treatment might be helpful in overall patterns of change and improvement.

For mindfulness practice, Kabat-Zinn [41] expresses it succinctly: ‘once a person has tasted the relaxation and calmness…these experiences become powerful motivators.’ (p.254). There is some qualitative evidence to suggest that participants who experience benefits from the course are more motivated to continue with their mindfulness practice [53]. Within the COM-B model, rewards can be considered as part of ‘Automatic motivation’, where positive emotions such as a rewarding experience arise through associative learning. Associative learning in this instance would take place when a student learns that practice brings benefits to their life (for instance increased awareness and concentration, discovering new ways to cope more effectively with current conditions).

Buddhist sources such as senior German Buddhist nun and teacher Ayya Khema, another teacher who brought mindfulness from Asia to the West, stresses the benefits of mindfulness, with statements such as ‘Why is mindfulness so important? I’d like to emphasise that mindfulness is not just something extra to be done in our spare time, but is essential for our wellbeing’ (Khema [44] p.9).

Both mindfulness and Buddhist literature discuss the rewards of practice as helpful in motivating and supporting practice. Langdon et al. [46], describe a ‘feedback effect of mindfulness practice, where its benefits seem to strengthen positive beliefs about it and so motivate people to practise more.’ (p279) This effect leads to a strengthening of the mindful attitude, or ‘being mode’, in daily life.

Noticing the benefits and enjoyment within practice was noted by several interviewees. Zoe ‘I really like the sitting practices, they make me feel connected with something other than, and it helps my thought processes if I feel a bit negative.’ (week 4). Eleanor found this too, saying post-course ‘I’m getting something from this so I’m going to stick with it’. Often course ‘graduates’ say that it is the benefits that have kept them going. It seems that either enjoying the practice, or the ‘off-cushion’ benefits, or rewards, can act as motivators.

What kinds of benefits did participants describe? Suzanne said after the course: ‘It’s beneficial – like a window opening up, a moment of real clarity, I am really here, like someone drawing back the curtains, and that’s such a nice feeling and that’s what I want to keep, what I aim for. Other benefits – I feel refreshed. When I’m not in the emotional up and down, I’ve had that calmer sense of being a bit more in control, how I want things to be’. Unsurprisingly, when the practice was more challenging, and the benefits less immediately obvious, participants tended to stop. This was true for Suzanne and Eleanor, who both hit difficult patches in their mindfulness practice post-course and found it impossible to maintain. However, both were keen to re-start by the time of the third interview, 4 weeks after the course had finished. Suzanne found after a while that ‘I kept getting angry, I knew there was past stuff and I thought maybe that’s coming out a bit. The practice was challenging at that time…maybe I needed some time to step away then come back to it.’ Zoe said at week 8 ‘Feeling peaceful and connected, I love it. If difficulties came up, I might find it more difficult to sit.’

Teachers can support participants in their practice by reminding them of, and highlighting, the benefits that they have noticed and how valuable they have been. They could also encourage participants to note these benefits down to support themselves in future times when motivation is low.

### Motivation: self-efficacy

Self-efficacy, defined as the belief in one’s ability to organise and execute the courses of action required to produce given attainments [7], has been widely investigated in health behaviours. Individuals with high self-efficacy are argued to be more conscientious, open to new experiences, and less emotionally reactive to stressors (for review, see [10]). Self-efficacy is one of the clearest correlates of health behaviours such as physical activity in adults according to a large-scale review of reviews [12]. Furthermore, there is experimental evidence that changes in self-efficacy can mediate the effects of a behaviour change intervention on increases in physical activity [20].

Self-efficacy is a core factor in many behaviour change models that try to explain and predict what factors influence people’s behaviour around their health (e.g., revised Health Belief Model; Protection Motivation Theory). Social cognitive theories also include self-efficacy and expectancy of the consequences of the behaviour, adding environmental factors as another determinant of whether the behaviour will be carried out or not [8, 9]. We suggest that self-efficacy enables and supports the discipline required for mindfulness practice. Within the COM-B model, self-efficacy can be considered as a component of Motivation [38], specifically motivation that requires reflection.

Mindfulness literature emphasises the importance of being disciplined: Kabat-Zinn urges us to be committed to the practice, and to stick with it for a period of time, while keeping a lightness as well [42]. In the Buddhist literature this is also emphasised by monk Bhante Gunaratana, a Sri Lankan who moved to West Virginia, U.S.A., founding a monastery and meditation retreat centre. He says ‘you never achieve anything great without effort…you must be willing to discipline yourself if necessary’ (Gunaratana [31] p.171). Resolve, or determination (Adhitthana parami), is also the eighth of the ten paramis or perfections that appeared in later Buddhist commentaries [5]. As Sucitto [68] describes, ‘this parami is then a foundation: intentions are pretty weak if one has no resolve to carry them out. You have to make the resolve to practise if you are to follow any path at all’ (p.153).

In the Pali Canon, the oldest written record of what the Buddha taught, argued to be authentic documentation [69], the Buddha encourages us to exert Right Effort in our practice - a practice of bringing enough effort, but not too much (“Samma Vayamo”) [5]. He explains this using the metaphor of tuning a stringed instrument so that the strings are neither too slack nor too loose. He implies that the practice will not happen by itself and that some application will be necessary; indeed, his last words were ‘Strive with earnestness!’ [2]. Yet he also cautions against applying excessive effort, which is similar to the ‘doing mode’ of ‘striving’, with its tendency to generate tension.

Our participant interviews illustrated the importance of self-efficacy. ‘What’s helped is my bloody-mindedness … if I approach something I decide I’m going to do it – it’s good and bad – it’s meant I sit down and do it, on the flip-side it’s a dodgy motivation at times, I want to improve and get results which isn’t really congruent with the approach’ (week 8). Georgie managed both to establish a regular practice during the course and maintain a level of practice that she was happy with after the course had finished. Her self-efficacy served her well, and she noticed that being stuck in ‘doing mode’ could be transformed into something more helpful, the discipline of a mindfulness practice that enabled her to simply ‘be’.

Self-efficacy is present in different people to different degrees (e.g., [40]). One course participant, Keith, had struggled with self-efficacy, but also found the course helpful in this regard: ‘Like a lot of good things… I procrastinated, even though I knew I wanted to do it. It’s like a self-destructive, self-defeating thing inside me. Maybe I have a pattern within … which says ‘I don’t know if you’re going to do that’…’ the message is based on an idea you have about yourself, an identity, over years of creating your identity of who you think you are, you’re not going to be able to do that. It’s been a source of great frustration; it’s held me back’ (week 4). By the end of the course, he reported that practice helped him in coping with self-defeating tendencies: ‘The self-defeating voice is still there. The crucial thing is I’m more aware of it than I was before. I can notice it, and as a result, I can make a positive change in the moment.’ (week 8). In the final interview 2 months after the course had finished, he said ‘I’m much more disciplined now than before the course’.

### How might we increase self-efficacy?

Higher levels of self-reported mindfulness have been associated with higher levels of self-efficacy [25]. While completing a mindfulness course may increase self-efficacy, there are other strategies such as using (i) social models – seeing peers successfully completing a task, (ii) social persuasion (iii) the experience of task completion [9]. We will look at each of these in turn.

First, we can consider the group as providing social models, where group participants can inspire and support each other. Most of the participants interviewed (by JM) commented on the supportive nature of the group. Second, social persuasion is present in our teaching: giving participants guidance to help them stay present during the practices, encouraging participants to practice at home, and highlighting instances when they have been mindful and the benefits that they have experienced. Kabat Zinn [41] emphasised the link between group setting and increasing self-efficacy, ‘Your self -efficacy can also increase if you are inspired by the examples of what other people are able to do.’ (p202).

Third, we suggest that the experience of completing mindfulness practice itself is important for participants in increasing their sense of self-efficacy. The commonly used phrase ‘as best you can’ in teaching is empowering as it helps participants to feel that what they can do is good enough. Williams and Penman [74] also seek to empower participants by saying ‘mindfulness is not complicated. Nor is it about ‘success’ or ‘failure’ (p.7). Participants are thus encouraged to continue, freed from the perceived threat of failure. This comes up frequently in the MBP classroom, and we believe it is important for teachers to emphasise that participants cannot fail. The Mindfulness-Based Interventions Teaching Assessment Criteria (MBI-TAC) encourages teachers to use phrases in the guided practices like ‘allowing experience to be as it is’, and avoiding language [like] ‘trying’ ‘working’ ([18] p.32).

A survey of health-care providers found that encouraging patients to record what they have achieved and to take pride in what they have accomplished were helpful strategies [51]. These strategies fit with MBPs and could be helpful in increasing a sense of mastery. Giving authority to the participants is also a part of empowering them and boosting their self-efficacy and confidence, and is a key facet of MBP teaching. As Segal et al. [64] describe: “Empowerment of participants is essential if they are to get the required amount of experience in using mindfulness. In the service of empowerment, learning should be based, wherever possible, on participants’ own experience rather than lectures from the instructor, and should embody the assumption that participants are the experts on themselves” (p.90).

Buddhist literature also seeks to foster self-efficacy, saying ‘don’t set goals for yourself that are too high’ ([32] p. 71). Many Buddhist books on mindfulness encourage beginners to start with short practices, not to worry when the mind wanders and so on. This is to ensure that beginners can gain some experience of mastery, rather than setting unrealistic goals, which may bring a feeling of failure. The importance of patience is also emphasised in Buddhist teaching (one of the seven attitudinal foundations also outlined by [41]. For example, Sucitto [68] emphasises patience, so that people do not have unrealistic expectations of quick results, which might lead to a sense of failure.

We suggest that teachers can help participants to believe in their ability to do the practice by (i) emphasising when they have been mindful, rather than when they have not (ii) encouraging them to be realistic in their practice schedule (iii) encouraging them to be guided by their own instincts (iv) letting them know that the practice is accessible for anyone but is not necessarily easy, so finding it difficult is not a sign of failure (v) guiding participants according to their tendencies and needs, either to become more disciplined or less driven.

### Motivation: participants’ beliefs about mindfulness practice

Participants’ beliefs about practice, how logical or credible it seems, and how much they consider it might work for them, is another factor that is likely to impact practice behaviour. The role of beliefs about the efficacy of the course of action, and about the threat of disease, appears in several models of behaviour change (e.g., the Health Belief Model and Theory of Reasoned Action and Protection Motivation Theory [59]. Further, there is a well-established phenomenon in clinical trials where participants do better if they find the treatment they are given plausible or credible [75]. If a treatment or intervention seems plausible, this can motivate participants to engage and there is evidence from other health domains for the efficacy of changing treatment beliefs on subsequent treatment-related behaviour. For example, one trial directly targeted beliefs about treatment for asthma using personalised messages and showed subsequent improvements in self-reported treatment adherence [57].

There is emerging evidence that participants’ beliefs about practice can influence their engagement. For instance, in one qualitative study, participants’ *belief* in the effectiveness of MBCT as an intervention for chronic pain influenced their commitment to practice [53]. Another recent study of MBCT demonstrated that participants’ beliefs about intervention outcomes predicted formal home practice completion [66]. Beliefs about the efficacy of the treatment can be viewed as part of the ‘Motivation’ component in the COM-B model.

Mindfulness literature encourages participants to approach the practice with an open mind, warning us not to try to influence what the exact outcomes will be [64]. Indeed, that open-minded, non-striving attitude is key to the approach. However, teachers are also encouraged to ‘Keep a balance between instructions to ‘let go’ of expectations (which can be de-motivating if overemphasised) and the willingness to believe that important changes may occur as a result of doing the mindfulness practice’ (p. 91 [64];). Fixed beliefs can also be detrimental; participants who have read articles in the press with titles like ‘Mindfulness changed my life’ can then spend time comparing their own experience to others’, usually unfavourably (see also Hopkins & Kuyken [35], for discussion of helpful and unhelpful upwards and downwards social comparisons in MBCT).

Buddhist literature propounds the benefits of mindfulness as a way of coping with the stress and difficulties of life, but rather than belief, the Buddha’s encouragement is for us to experience the benefits of the teachings for ourselves (Kalama Sutta, [1]). The Buddha offered the Eightfold path, which includes mindfulness, as the course of action to alleviate stress. Indeed, mindfulness is found throughout the wide scope of the Buddha’s teachings towards a life free from stress. The Buddha explains that ‘faith is the seed’, indicating that faith can precede direct knowledge of the benefits of practice [3]. Faith (also translated as ‘confidence’ or ‘trust’), does not stop there; it increases as we see the benefits and our confidence in the practice grows. This happened with Eleanor: ‘I’m getting something from this so I’m going to stick with it’ (quoted previously, post course).

Sucitto [68] explains the difference between faith and belief: ‘With faith, the energy is an opening of the heart, whereas belief closes the mind by locking it onto an idea or theory. Belief employs energy to defend or attack, and not to enquire. Faith, on the other hand, always benefits from enquiry. When you place faith in someone or something, it means you will give them clear attention and take seriously what they say. But the Buddha emphasises that such a faith has to be backed by investigating the truth and working with it in yourself’ (p.102).

Belief motivated Noeleen: she was suffering from depression and her counsellor advised her to do a mindfulness course. Her suffering was a motivation for her to engage in practice, although she reported not experiencing any benefits in the first interview: ‘A sense of desperation, I have to do something about the state that I’m in, that nothing else had worked and this was the last chance to find something that helped me…my counsellor said that she had heard mindfulness could help depression’ (week 4). Liam, on the other hand, believed that mindfulness would help him, but that belief alone was not enough to motivate him to establish a regular practice; he lacked the urgency that Noeleen had, saying ‘I have a belief it will help me but somehow I don’t get round to it’ (week 8).

Suzanne demonstrated this open-minded attitude in the post-course interview: ‘I wasn’t sure if it would be something that would work for me – it might be ok for some people and for others it might not be, and the only way I’ll find out is to try it and see what unfolds. I didn’t want to come in with preconceived ideas about it.’

Most course participants have direct experience of stress or low mood in their lives. They typically arrive at the course with some belief that mindfulness can help them and have self-selected to participate. As participants start to experience these benefits, or hear about them from fellow participants, belief may become less necessary. As Kabat-Zinn [41] explains, ‘you have to actually practice mindfulness in order to reap its benefits and come to understand why it is so valuable.’ (p21).

Belief in the practice, alongside participants’ intentions and commitment, can often grow with time and experience, though many come to the course with some belief that it can help, either from a health care professional, a friend or from reading. Belief in a treatment may be important initially for participants to arrive at mindfulness training and engage with practice. We suggest that belief will be superseded by experiencing the benefits of practice. We argue that fixed ideas about benefits can even become an obstacle, as they can stand in the way of experience. As teachers, we need to encourage confidence in the practice, but balance that with open-mindedness about how it will unfold for each person. We may do that with reference to books and studies, personal experience [50], and as the course goes on, by giving participants the opportunity to discuss and share the benefits they have experienced, hear from others, and by reminding participants of the benefits they have noticed as well as normalising that these will vary.

### Motivation: participants’ interest in self-care

Self-care can be defined as the ability to promote, maintain health, prevent disease and cope with illness, with or without the support of a health care provider [76]. The WHO suggests that supporting self-care behaviours requires systematic action, including raising awareness, changing beliefs and building skills. These actions may all be targeted within an MBP. There is emerging evidence suggesting that health-promoting preventative self-care interventions show promise in increasing the well-being of healthy people [56]. For chronic illness, self-care interventions have a more established evidence-base [58]. Participants’ interest in self-care can be categorised as ‘Motivation’ within the COM-B model, again as a reflective process that can direct the behaviour.

Buddhist literature and mindfulness literature emphasise a caring, heart-based approach, which is absent in many of the health-psychology models. Kabat-Zinn [41] was clear on the nourishing power of practice, saying that ‘making time for formal practice every day is like feeding yourself every day. It is that important.’ (p.431). Williams and Penman [74] suggest taking on the practice ‘as a strong commitment to yourself’. Expanding on practical ways to encourage interest in self-care, Segal et al. [63] suggest ‘link[ing] the maintenance of the practice with something about which they care deeply’ (p.302), giving the example of a woman who felt more available for her children when she practised: an interesting expansion on the idea of self-care. The potential of mindfulness as an act of self-care will only become apparent as participants start to experience the benefits, although some participants enjoy it right from the first mindfulness exercise with the raisin (The Raisin Meditation).

Suzanne, at week 4, described her practice as ‘Having that time to think about me and what I want to do – being kind to myself…. I feel like it’s important, an investment, for me, effectively…to look after myself…’ After the course had finished, she said ‘I have a strong intuition that this is something that is good for me’; that intuition and desire to care for herself kept her going with a regular practice. Motivation to care for oneself is not always easy, as Liam noted at week 4: ‘There’s definitely a theme of not being that kind to myself, that’s something that I’m doing mindfulness for’. It would be helpful for a teacher to look out for this tendency and offer extra support and encouragement and reminders that self-care is a healthy motivation.

The motivation to care for oneself can be self-reinforcing and is a major theme throughout MBPs. As people learn to bring a self-care attitude to themselves, they are more likely to prioritise practice as they experience its benefits. This is one factor where participants expressed noticing change, realising how hard they could be on themselves, and learning, as Eleanor said at week 8 – [to be] ‘a bit gentler on myself now’ as well as finding that regular practice is an excellent way of caring for themselves, as Suzanne explained at week 4: ‘I need to keep in mind that this is beneficial for me … having that time to think about me and what I want to do – being kind to myself.’

To cultivate motivation within MBPs, we might focus on the positive reasons for engaging in mindfulness practice, such as having greater present moment awareness. In this way, we might also change beliefs about practice. Focusing on the practice as a way of caring for oneself, a major theme in MBPs, is a valuable way to help people to practice. Emphasising the self-care aspect can also reduce the feeling that practice is a chore. We suggest that those expressing low self-care motivation may particularly need encouragement and support.

## Conclusions

Both MBSR and MBCT emphasise daily mindfulness practice, both formal and informal, as a core component of the programme. Our understanding as teachers and researchers of how best to support and guide participants doing these courses is at an early stage. In this paper, we present seven factors that we believe are important in establishing and maintaining a regular mindfulness practice during an 8-week mindfulness course. These include self-efficacy, planning, self-care, beliefs about mindfulness practice and social support, the relationship with the teacher and experiencing benefits of practice. In selecting these factors, we draw upon and integrate literature from health behaviour change (particularly from physical exercise, acknowledging that mindfulness practice and exercise are not perfectly analogous), mindfulness and Buddhist literature and participant interviews. We argue that some of these factors can be directly influenced by mindfulness teachers and we make a number of practical suggestions as to how we can do this.

**Fig. 1:**
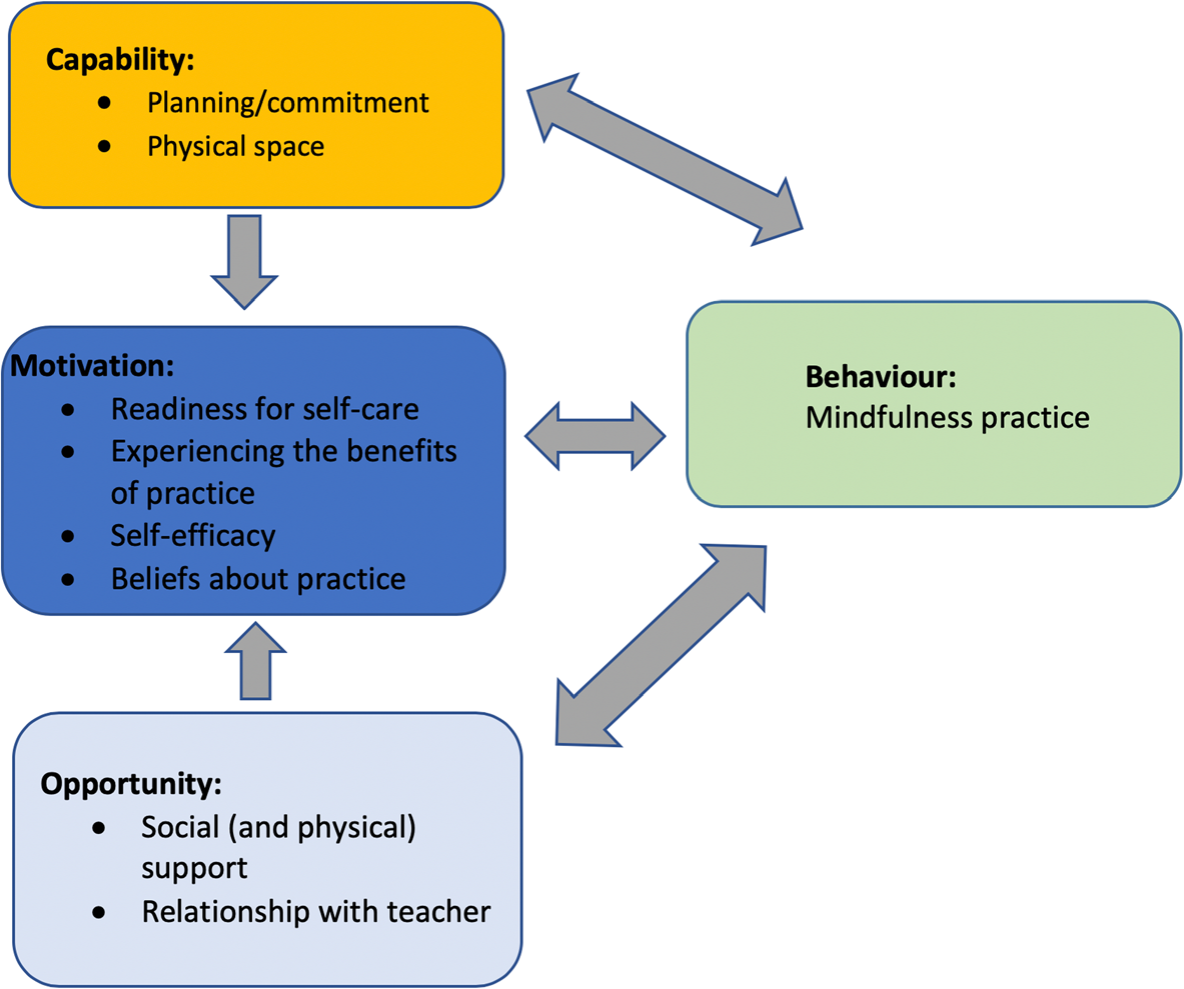
We use the ‘COM-B’ (Capability, Opportunity, Motivation - Behaviour) system to categorise the factors that we suggest may be important for establishing a mindfulness practice [52]. The single and double arrows represent potential influence between factors. For instance, opportunity (social support) can influence motivation, as can self-efficacy. Practicing mindfulness can alter capability, motivation and opportunity

We consider a number of these factors to be ‘necessary’ for establishing a mindfulness practice: self-efficacy, experiencing the benefits of practice, and wanting to care for oneself (see Fig. 1). These three factors map onto Motivation component of the COM-B model, which has a central role in the intersection between Capability and Opportunity. Self-efficacy, when combined with growing experience and confidence in the benefits of the practice and one’s ability to ‘do it’, can generate commitment to practice as a way of caring for oneself. We suggest that if a participant does not have the self-efficacy to establish a practice in the first place, they will not experience its benefits. If the benefits either do not appear or are not sufficiently positive, the participant may not be motivated to practice. If the participant does not have enough motivation for self-care, they will not pursue these benefits. We argue that all three factors are necessary, and none alone are sufficient. These three factors, and their relative importance, could be tested in future empirical studies.

Other factors, such as social support, the relationship with the teacher, (both related to Opportunity) participants’ beliefs (Motivation) and their planning/commitment (Capability) may interact in complex ways. For example, the COM-B model indicates that Capability (planning/commitment) impacts on Motivation, and both can impact on the behaviour, practice. For example, if a participant has difficulties planning a time to practice, this will impact on Motivation-related factors, such as the likelihood of experiencing the benefits of practice. Similarly, Opportunity will impact on Motivation: if a participant has sufficient social support to practice, they are more likely to be motivated to do so. This also highlights the importance of the orientation/screening process to assess whether a prospective participant has the capability to engage with the practice and the course.

These factors will be relevant in a different combination for each participant and may change with time. It is our experience that participants vary widely in what they find helps them establish and maintain a practice; some need a group; others have sufficient self-efficacy to continue more or less alone. These factors may change in importance within the individual over time. Here, we highlight factors that may be helpful for teachers to bring skilfully and appropriately into their teaching and invite participants to reflect on what would be the best support for them at particular junctures.

While we have focused primarily on the ‘Motivation’ component, the COM-B model also places an individual’s Capability and Opportunity as crucial in understanding a target behaviour. These are clearly important, but participants who enrol in an MBP may already have a certain level of Capability and Opportunity. While we have not discussed physical capability, it is understood that many arrive with certain difficulties like pain that might impact on their practice behaviours. We suggest that the COM-B model provides a useful framework to consider the multiple interacting factors that impact on practice behaviour. It provides a theory-driven approach for teachers to look at the different overarching components that will impact whether participants will establish a mindfulness practice. We acknowledge, however, that there are many models of health behaviour change that we have not considered here (for a recent review, see [54]). Finally, we also note that factors such as quality of practice may be important in promoting learning and positive change [21], but we do not explicitly address how to improve practice quality here.

## Data Availability

Data sharing is not applicable to this article as the interviews were not fully transcribed during the current study.

## Abbreviations

MBPs: Mindfulness-Based Programmes
COM-B: Capability, Opportunity, Motivation and Behaviour model
MBCT: Mindfulness-Based Cognitive Therapy
MBSR: Mindfulness-Based Stress Reduction
